# Physicochemical parameters and nutritional profile of back and abdomen muscle of fresh golden pompano (*Trachinotus ovatus*) and hybrid grouper (*Epinephelus lanceolatus × Epinephelus fuscoguttatus*)

**DOI:** 10.1002/fsn3.3139

**Published:** 2022-11-14

**Authors:** Ahtisham Ali, Jinfang Wang, Imran Khan, Shuai Wei, Qinxiu Sun, Qiuyu Xia, Zefu Wang, Zongyuan Han, Shucheng Liu

**Affiliations:** ^1^ Guangdong Provincial Key Laboratory of Aquatic Products Processing and Safety, Guangdong Province Engineering Laboratory for Marine Biological Products, Guangdong Provincial Engineering Technology Research Centre of Seafood Key Laboratory of Advanced Processing of Aquatic Product of Guangdong Higher Education Institute, College of Food Science and Technology, Guangdong Ocean University Zhanjiang China; ^2^ Southern Marine Science and Engineering Guangdong Laboratory Zhanjiang China; ^3^ Department of Food Science and Technology The University of Haripur Haripur Khyber Pakhtunkhwa Pakistan; ^4^ Collaborative Innovation Centre of Seafood Deep Processing Dalian Polytechnic University Dalian China

**Keywords:** chemical composition, golden pompano, hybrid grouper, nutritional profile, physical parameters

## Abstract

Golden pompano (*Trachinotus ovatus*) and hybrid grouper (*Epinephelus lanceolatus × Epinephelus fuscoguttatus*) has widely been distributed in China and Southeast Asian countries with great commercial importance. In this study, the nutritional profiles, chemical and physical parameters of back and abdomen muscles were determined. Significantly different (*p* < .05) proximate compositions were found in two fish muscles. The contents of water‐soluble protein, salt‐soluble protein, and non‐nitrogenous protein were higher in the golden pompano while salt‐insoluble proteins were higher in the hybrid grouper. The main minerals found were K (3700.56–4495.57 μg/g) followed by P > Na > Mg > and Ca, respectively. Fatty acids contents consisted of polyunsaturated fatty acids ranging from 29.40% to 43.09% and saturated fatty acids 28.33% to 39.61%. The muscles were rich in n‐3 PUFAs with n‐6/n‐3 ratio of 1.36%–2.96% in the back and abdomen. On the other hand, total amino acid and non‐essential amino acid contents were found higher in the hybrid grouper while essential amino acid and delicious amino acid contents were higher in the golden pompano. Glutamic acid was the most predominant amino acid. The amino acid scores (AAS) of six amino acids were close to 1.00, whereas lysine showed the highest AAS while tryptophan was the most limited essential amino acid in all muscles, respectively. These results indicated golden pompano and hybrid grouper exhibited a varied nutritional composition and offered a good nutritional profile.

## Graphical abstract

Golden pompano (*Trachinotus ovatus*) and hybrid grouper (*Epinephelus lanceolatus × Epinephelus fuscoguttatus*) has widely been distributed in China and Southeast Asian countries with great commercial importance. In this study, their nutritional profiles, chemical and physical parameters of back and abdomen muscles were determined. These results indicated golden pompano and hybrid grouper exhibited a varied nutritional composition and offered a good nutritional profile.

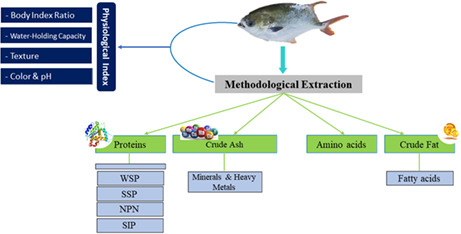

## 1 INTRODUCTION

People around the world are paying increasing interest to the accessibility of healthy food aimed at improving human health, reducing the incidence of diseases, and providing nutritional benefits (Chen et al., 2022). In this regard, marine fish are widely produced in many parts of the world and are renowned for their high nutritional value because of their high protein contents, essential amino acids, low fat, minerals, and a good source of polyunsaturated fatty acids, especially eicosapentaenoic acid (EPA) and docosahexaenoic acid (DHA) (Byrd et al., 2021).

Fish muscle has been considered the main edible part of fish and its major component is the protein which determines the nutritional value of fish muscle (Ali et al., 2021). Fish is a rich source of n‐3 polyunsaturated fatty acids (n‐3 PUFAs) including EPA and DHA which offer potential health benefits involved in reducing the risk of cardiovascular diseases, facilitating the proper functioning and development of the brain, visionary receptors, and the reproductive system (Delpino & Figueiredo, 2021). Furthermore, EPA and DHA are predominantly known as the precursors of composite hormones, i.e., eicosanoids, which have been involved in various metabolomic processes of the human body (Inhamuns & Franco, 2008). The optimum amounts of essential fatty acids, essential amino acids (EAA), PUFAs/saturated fatty acids (SFA), and n‐3 PUFAs/n‐6 PUFAs ratios are responsible to evaluate the indexes of the nutritional quality. However, the nutritional composition of fish is mostly influenced by the following factors such as species, sexual maturity, fish size, age, season, water quality, and feeding system (Marques et al., 2021).

Golden pompano (*Trachinotus ovatus*) and Hybrid grouper (*Epinephelus lanceolatus × Epinephelus fuscoguttatus*) are marine species that belong to the *Carangidae* and *Serranidae* families that are famous for their great commercial importance, which has widely been distributed in China and Southeast Asian countries. China has contributed 81% (while mainland 65% and Taiwan 17%) of total grouper production worldwide (Rimmer & Glamuzina, 2019). They are popular and have more economic value due to their high nutrition, fast growth, pleasant taste, high tolerance to stress, and increasing market demands (Rimmer & Glamuzina, 2019). Unfortunately, insufficient information is available regarding their proximate composition and nutritional composition.

Therefore, this present study aimed to investigate the nutritional value of golden pompano and hybrid grouper fish and comparatively analyzed the differences between the back and abdomen muscles regarding their proximate composition, protein contents, texture, color, minerals, fatty acids, and amino acid composition. To the best of our knowledge, this current study is the first of its kind to investigate the nutritional composition of the back and abdomen muscles.

## 2 MATERIALS AND METHODS

### 2.1 Physical index

**FIGURE 1:**
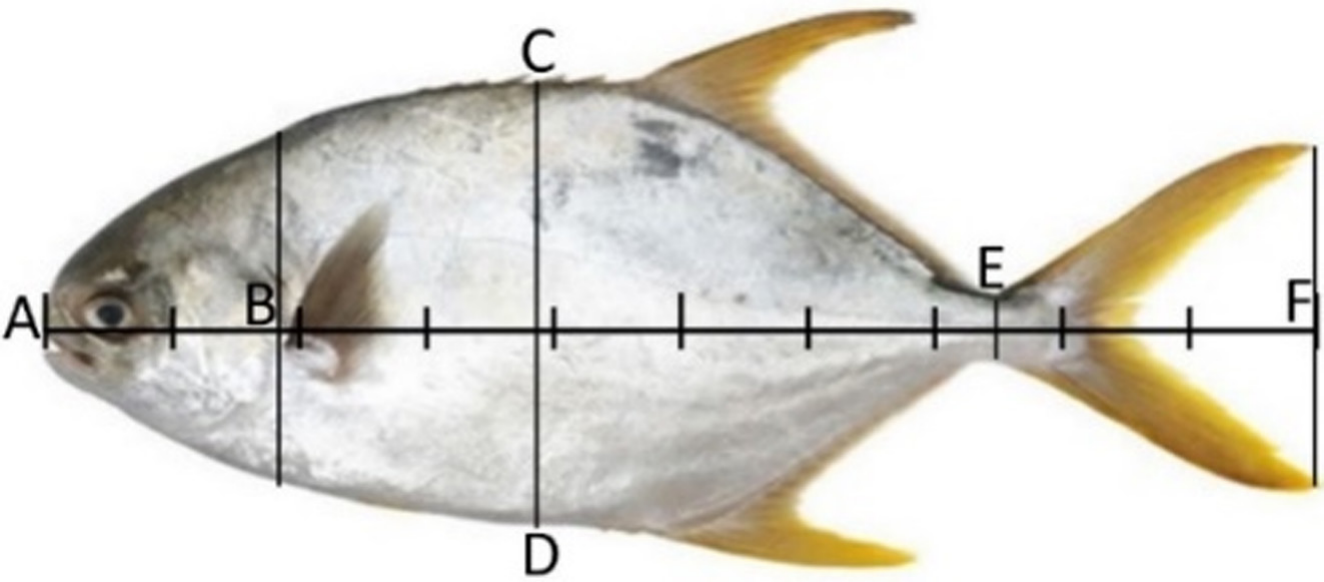
Body index of fresh golden pompano

The body index of the fresh fish was determined. The total length (A‐F), body length (A‐E), head length (A‐B), tail length (E‐F), and body height (C‐D) of the sample was calculated according to the scale as shown in Figure 1. The fat fullness of the fish was calculated according to the formula:

(1) $$\mathrm{Fat}\text{fullness}=\frac{\text{Total weight}\left(g\right)}{\text{Total length}\left({\mathrm{cm}}^{3}\right)}\times 100$$

The body mass of marine fish was determined to separate the scales, fins, internal organs, skin, heads, bones, and flesh of fish, and weighed separately. The percentage of body mass is the mass fraction of each part.

### 2.2 Proximate composition

The moisture content was measured by using the oven‐drying process at 105°C. The crude protein content was determined by the micro‐Kjeldahl method while crude fat contents were investigated by the Soxhlet extraction method. The crude ash contents were measured by ignition in a muffle furnace at 55°C.

### 2.3 Texture determination

Texture profile analyses of fresh muscles were determined by using a Texture Analyzer (TA‐XTplusC, Godalming) equipped with a 1 kg cylindrical probe with slight modifications (Sung et al., 2019). The sample cut approximately 1.5 × 1.5 × 1.0 cm was compressed vertically by using a 5 mm cylinder‐shaped probe (P – 5). The test condition involved two simultaneous cycles of 25% shearing force at a pretest speed of 1.0 mm/s, a test speed of 1.0 mm/s, and a posttest speed of 1.0 mm/s with an initiation point of (0.05 N). Texture profile including hardness, cohesiveness, gumminess, adhesiveness, springiness, and chewiness was examined in triplicate.

### 2.4 Color and pH determination

The color profile of fish muscle was determined by a colorimeter (CR‐20, Konica Minolta Inc.). L* (lightness), a* (redness/greenness), and b* (yellowness/blueness) values were determined, and whiteness was calculated as follows:

(2) $$\text{Whiteness}=\sqrt{100-\left[{\left(100-L^{*}\right)}^{2}+{a^{*}}^{2}+{b^{*}}^{2}\right]}$$

Fish muscle pH was measured by weighing 10 g of fresh fish muscle and then homogenized in 100 ml of deionized water at 5000 rpm for 60 s at 4°C. The pH value was measured in duplicate with a pH meter (FE‐28, Mettler Toledo).

### 2.5 Water‐holding capacity

#### 2.5.1 Cooking loss

For the cooking loss, the sample of fresh muscle (15 × 15 × 10 mm) was wrapped in a polyethylene bag and then dipped in a water bath at 75°C for 10 min. The cooking loss rate was calculated based on the initial weight (*W*_*i*_) and final weight (*W*_*f*_) as follows:

(3) $$\text{Cooking loss}\left(\%\right)=\frac{W_{i}-W_{f}}{W_{f}}\times 100$$

#### 2.5.2 Drip loss

For the drip loss, all the samples were weighed and packed in a polyethylene bag before freezing. Samples were removed from their bag, thawed, and weighed. Special care was taken to collect the drip loss physically present on the surface of the sample using filter paper. The drip loss was calculated based on the initial weight (*W*_*i*_) and final weight (*W*_*f*_) of the samples according to the following equation:

(4) $$\text{Drip loss}\left(\%\right)=\frac{W_{i}-W_{f}}{W_{f}}\times 100$$

#### 2.5.3 Water loss

The water loss of fresh fish muscle was examined as follows. Briefly, approximately 2 g of sample was added in the plastic tube and centrifuged (Sigma, 3‐30KS) at 1760 g for 10 min at 4°C. The water loss was calculated based on the initial weight (*W*_*i*_) and final weight (*W*_*f*_) by using the following equation:

(5) $$\text{Water loss}\left(\%\right)=\frac{W_{i}-W_{f}}{W_{f}}\times 100$$

#### 2.5.4 Low‐field nuclear magnetic resonance (LF‐NMR)

The transverse relaxation data (T2) were determined by using nuclear magnetic resonance (NMR) analyzer (Suzhou Niumag Analytical Instrument Co.). Magnetic field strength (0.5 T) was employed with a permanent magnet. The Carr–Purcell–Meiboom–Gill (CPMG) system parameters were arranged as 180° and 90° pulses while π‐values (21, 42, and 200 μs) were used to measure the relaxation results. Water contents were removed from the sample surface before putting it into the 60 mm diameter of the NMR tube and then observed the sample under 10,000 echoes in triplicate. CPMG data inversion was measured using MultiExp Inv analysis software to collect different components, T2‐distributed curve, and relaxation parameters.

### 2.6 Electron microscopic determination

The production of paraffin slices determined by the muscle sample used to trim into 0.5 cm × 0.7 cm × 0.5 cm rectangular small pieces. Immediately, the muscle pieces were placed in 4°C Carney's fixative solution (glacial acetic acid: chloroform: absolute ethanol = 1: 3: 6, v/v) and set at low temperature for 24 h. After fixation, dehydrated with graded ethanol (50% ethanol for 30 min, two times; 75% ethanol for 30 min; 85% ethanol for 30 min; 95% ethanol for 30 min; and 100% ethanol for 30 min, 2 times) and then put in a volume ratio of 1:1. Transparent in the mixed solution of ethanol and xylene for 1 h and then put in the xylene solution for 1 h, two times until there is no white spot in the center of the sample.

After the transparency was completed, the sample was soaked in a mixture of xylene and paraffin solution with a volume ratio of 1:1 for 30 min (62–65°C) two times, and finally, into the paraffin wax soaked for 2 h, two times. The embedding treatment was carried out after the immersion: slightly heated the tweezers and metal‐embedding mold, then used the warm tweezers to take out the sample holder, placed it in the center of the embedding plate, and reversed the embedding class to the metal‐embedding mold. Pour the molten paraffin liquid along the gap of the embedding plate and finally remove the paraffin block after cooling. The paraffin block was trimmed into a square and put into the microtome. The slice thickness was 10 μm and slice angle was 10°. The completed slices were placed in a 45°C constant temperature water bath to unfold the slices. Then, the slides were placed in an electrothermal thermostatic blast dryer (65°C) to dry for 2 h. The dried slides were dewaxed in xylene for 10 min, two times, and then washed with graded ethanol (100% ethanol for 10 min, two times; 95% ethanol for 5 min; 85% ethanol for 5 min; 75% ethanol for 5 min; 50% ethanol for 5 min, two times; and distilled water for 5 min, two times) to remove xylene. After dewaxing, the samples were stained with HE, stained with hematoxylin for 5 min, washed with water for 10 s, stained with eosin for 2 min, and washed with water for 1 min. After dyeing, it was naturally dried, sealed with neutral gum, and finally, subjected to microscopic observation.

### 2.7 Protein fractionation

Fresh fish muscles were used for extracting protein fractions. Briefly, approximately 3 g of fresh muscle sample was added to 30 ml phosphate buffer solution (15.6 mM Na_2_HPO_4_ and 3.5 mM KH_2_PO_4_) and homogenized (IKA, T18 DS25) for 1 min at 10,000 rpm at pH 7.5. Then, the homogenate was centrifuged at 4°C at 10,509 g for 10 min using a Sigma 3‐30KS centrifuge. The supernatant was collected in a separate 50 ml Falcon tube and to the residue added another 30 ml phosphate buffer solution and pass‐through similar homogenization and centrifugation processes. Both supernatants were combined and added to 5% trichloroacetic acid. Afterward, the above‐prepared solution was centrifuged at 10,509 g for 15 min and the precipitate was referred to as water‐soluble protein and the filtrate was collected as a nonnitrogenous protein fraction. The precipitate obtained above was homogenized (10,000 rpm, for 1 min) in 30 ml of phosphate buffer (15.6 mM Na_2_HPO_4_ and 3.5 mM KH_2_PO_4_) comprising 0.45 M KCL, pH 7.5, followed by centrifugation at 10,509 g for 20 min to collect the supernatant. The above process was repeated two times. The above‐obtained supernatants were combined and used as the salt‐soluble protein fraction. The precipitate found at this stage was treated with 30 ml of 0.1 N NaOH and homogenized for 2 h followed by centrifugation at 10,509 g for 15 min at 4°C. The resultant supernatant obtained at this stage was determined as the salt‐insoluble protein. The final filtrate was collected as a stroma segment. The protein contents of each fraction were determined by using the Coomassie brilliant blue commercial assay kit (Beijing Leagene Biotechnology Co., Ltd).

### 2.8 Mineral and heavy metal contents

For the mineral contents, 0.3 g of abdomen and back muscle was weighted in a digestion vessel and dissolved in nitric acid (12 ml; 65% v/v), respectively. The resulting solution was initially digested in a vessel at 130°C for 10 min followed by second digestion at 150°C for 5 min, and final digestion was performed at 180°C for 45 min. The digested samples were allowed to cool and thereafter made up the volume up to 25 ml with nitric acid (1%, v/v) solution and filtered through a 0.22 μm membrane filter. The following elements calcium (Ca), sodium (Na), magnesium (Mg), phosphorus (P), potassium (K), zinc (Zn), iron (Fe), copper (Cu), selenium (Se), manganese (Mn), and lead (Pb) were investigated using coupled plasma mass spectrometer (Perkin–Elmer, Model 4300 DV, Norwalk).

### 2.9 Fatty acid analysis

Fatty acids were determined using a chloroform–methanol (2:1 v/v) solvent system containing 0.05% butylated hydroxytoluene (BHT). The transesterification of the fatty acids in the muscles was conducted by acidic methylation to fatty acids methyl esters (FAME). The derived fatty acids methyl esters (FAME) were then determined using gas chromatography (Thermo Scientific Inc., Trace GC Ultra‐2008) equipped with flame ionization detector (GC‐FID) and a DB‐WAX‐fused silica capillary column (50 m × 0.20 mm × 0.20 μm film thickness, J&W Agilent Technologies, Inc.). The chromatographic conditions were as follows: injection temperature 230°C; initial oven temperature was set at 85°C for 2 min; then gradually programmed to 180°C for 5 min at a rate of 5°C/min, respectively. The oven temperature was then increased to a final temperature of 240°C at 10°C/min and seized for 5 min. Nitrogen gas was employed as the carrier gas at a constant flow rate of 1 ml/min. The fatty acids compounds were analyzed by comparison of their retention time and peaks area with known standards (SupelcoTM 37 Component FAME Mix). All data were analyzed in triplicate values.

### 2.10 Amino acid analysis

The amino acid composition was measured by using a high‐speed Amino Acid Analyzer (L‐8900, Hitachi High‐Technologies Co.). Approximately, 30 mg of fish muscle sample was weighed in a screwed glass which contained a translucent‐based blue silicone septum sealed. For digestion, the muscle sample was added 5 ml of 6 N HCl solution in a nitrogen‐sealed tube and then immersed in a sand bath at a temperature of 110°C for 24 h, respectively. The cooled digested sample was then washed in a 50 ml volumetric flask with ultrapure water. One ml of the above solution was added into a 4 ml ampoule bottle and then evaporated in a rotary evaporator (IKA RV10). The sample was re‐suspended in a 1 ml of 0.02 N HCl solution, filtered with a 0.22 μm membrane using a hydrophilic polyethersulfone filter for removing any impurity and residues. After the filtration process, a 20 μl solution was used to determine the amino acid contents using the high‐Speed Amino Acid Analyzer equipped with Hitachi ion‐exchange resin‐packed column (4.6 mm × 60 mm and the particle size 5 μm). A coloring solution ninhydrin was primarily used as a reactive reagent for the analysis of amino acids. The data found were analyzed as mg/g performed in triplicate.

The nutritional value of golden pompano muscle was analyzed according to the Food and Agriculture (FAO)/World Health Organization (WHO) amino acid reference pattern. The EAA score and essential amino acids index (EAAI) were calculated by using the following Equations 1 and 2, respectively:

(6) $$\mathrm{EAA}=\frac{\mathrm{mg}\text{of}\mathrm{EAA}\text{in}1g\text{of the test protein}}{\mathrm{mg}\text{of}\mathrm{EAA}\text{in}1g\text{of}\mathrm{egg}\text{protein}}\times 100$$

(7) $$\text{EAAI}=\sqrt{\frac{\left(\text{EAAI}\times 100\right)\left(\mathrm{EAA}2\times 100\right)\left(\cdots \right)\left(\text{EAAn}\times 100\right)\left[\text{sample}\right]}{\left(\text{EAAI}\times 100\right)\left(\mathrm{EAA}2\times 100\right)\left(\cdots \right)\left(\text{EAAn}\times 100\right)\left[\text{reference}\right]}}$$

The amino acid score was calculated using the following equations:

(8) $$\mathrm{AAS}=\frac{\text{Amount of amino acid}\mathrm{per}\text{test protein}\left(\mathrm{mg}/g\right)}{\text{Amount of amino acid in}\mathrm{FAO}/\mathrm{WHO}\text{reference}\left(\mathrm{mg}/g\right)}$$

(9) $$\mathrm{CS}=\frac{\text{Amount of amino acid}\mathrm{per}\text{test protein}\left(\mathrm{mg}/g\right)}{\text{Amount of amino acid in}\mathrm{egg}\text{protein}\left(\mathrm{mg}/g\right)}$$

### 2.11 Statistical analysis

The analytical data were carried out in three replications using samples from golden pompano muscles. The data were analyzed by using a one‐way analysis of variance (anova) followed by Tukey test using SPSS software version 26.0 (IBM Corp.). The threshold of statistical significance was set to a *p* < .05.

## 3 RESULTS AND DISCUSSION

### 3.1 Physical index

The body index and mass ratio of golden pompano and hybrid grouper between different muscle types as shown in Table 1. The ratio of body length to height of the hybrid grouper was 1.76%, which was higher than the golden pompano (1.41%), while the ratio of body mass/total length in the golden pompano was higher than that of the hybrid grouper. Similarly, the muscle mass/body mass ratio of the golden pompano was significantly higher than the hybrid grouper. Overall, the golden pompano fat fullness value (3.89 g/cm^3^) was greater than the hybrid grouper (1.67 g/cm^3^). These values were higher than seabream (2.79 g/cm^3^) (Alomar et al., 2021).

**TABLE 1:** Physical index of fresh golden pompano and hybrid grouper (weighed 500–1000 g)

Index	Golden pompano	Hybrid grouper
Head length/Total length (%)	20.36 ± 1.28	28.26 ± 1.26
Tail length/Total length (%)	22.99 ± 1.97	20.02 ± 0.66
Trunk length/Total length (%)	56.64 ± 2.26	51.45 ± 1.53
Head length/Body length (%)	26.45 ± 1.73	55.03 ± 3.92
Body length/Height (%)	1.41 ± 0.10	1.76 ± 0.14
Body mass/Total length (g/cm)	17.1 ± 2.01	17.08 ± 0.32
Head mass/Body mass (%)	23.15 ± 2.91	29.00 ± 0.74
Tail mass/Body mass (%)	1.89 ± 0.20	1.76 ± 0.24
Trunk mass/Body mass (%)	74.94 ± 3.01	50.67 ± 0.78
Muscle mass/Body mass (%)	49.83 ± 4.01	33.16 ± 1.82
Visceral mass/Body mass (%)	5.99 ± 1.15	8.79 ± 0.60
Skin scale mass/Body mass (%)	5.50 ± 0.43	8.90 ± 1.23
Bone mass/Body mass (%)	10.92 ± 1.87	8.96 ± 0.49
Gill mas/Body mass (%)	1.17 ± 0.52	1.05 ± 0.09
Fin mass/Body mass (%)	1.51 ± 0.17	3.18 ± 0.24
Fat fullness (g/cm^3^)	3.89 ± 0.36	1.67 ± 0.12

*Note*: The data were determined as means ± SD from triplicate values. Fat fullness (g/cm^3^) = total weight (g)/total length (cm^3^) × 100. Body length/height (%) = body length (cm)/body height (cm). Body mass/total length (g/cm) = total body mass (g)/total body length (cm).

### 3.2 Proximate composition

**FIGURE 2:**
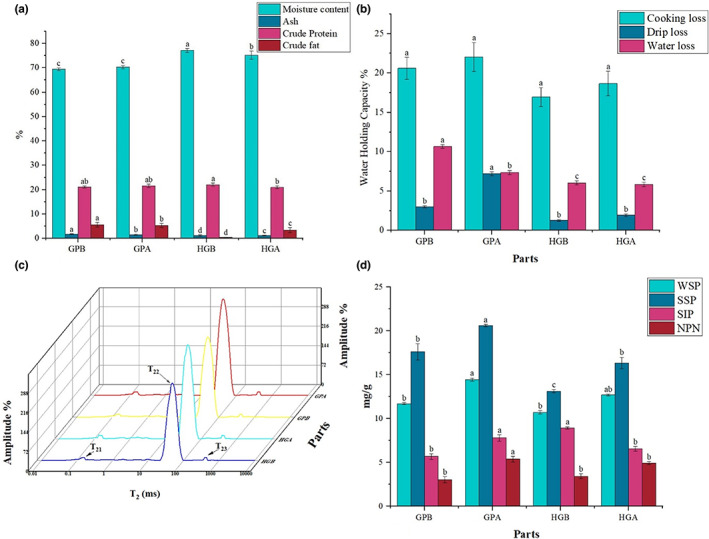
The proximate composition (a), water‐holding capacity (b), the transverse relaxation time T_2_ (c), and protein compositions of back and abdomen muscles of fresh golden pompano and hybrid grouper. The data were determined as means ± SD from triplicate values. Different letters indicate a significant difference between the back and abdomen muscles (*p* < .05). GPB and GPA (golden pompano); HGB vs. HGA (hybrid grouper).

The proximate composition of the back and abdomen muscles from golden pompano and hybrid grouper was investigated as shown in Figure 2a. Generally, significant differences (*p* < .05) were found in all proximate compositions of different parts of the fish muscle. This result was in accordance with the pirarucu (*Arapaima gigas*) muscle (Chyuan‐Yuan et al., 2018). Moisture was considered as the main constituent in all parts of the muscle. The highest contents were obtained in the hybrid grouper muscles (75.22%–77.16%) followed by golden pompano (69.43%–70.32%). Crude protein, fat, and ash were found in the ranges 20.92%–22.01%, 0.45%–5.64%, and 1.18%–1.77% in the back and abdomen muscle of each fish.

The moisture contents found in both fish muscles were generally higher than in Florida pompano (Novriadi et al., 2019). Generally, fish contained total protein contents in the ranges 11%–24% (wet weight). Protein was found as a second major constituent that ranged from 20.92% to 22.01% in all muscles. The hybrid grouper had higher protein contents than the golden pompano (*p* < .05), especially the abdomen muscle. Our findings agreed with the previous results where the abdomen muscle had higher crude protein contents than the back muscle (Martins et al., 2017). Whereas, the higher protein contents were investigated in the back muscle of *D. Labrax* (19.5% and 17.5%) and *O. mykiss* (20.3% and 19.3%), respectively Testi et al., 2006. The differences in the protein contents could be related to species, sex, state of nutrition, age, environment, and feeding (Hlordzi et al., 2022). The protein contents in our study were higher than the *T. carolinus* (19.5%) (Novriadi et al., 2019).

Furthermore, the crude fat contents were higher in golden pompano, especially back muscle. Golden pompano back muscle had an approximately five times higher crude fat content than the hybrid grouper back muscle. In hybrid grouper, the abdomen contained approximately 1.92 times higher crude fat contents than the back. The content of crude fat in hybrid grouper followed the same pattern that the abdomen muscle was particularly rich in chromoproteins and possessed about two to five times more crude fat than the back muscle (Chaijan et al., 2013). But this statement was not true for the golden pompano in which the back contained slightly higher crude fat contents than the abdomen muscle. This was probably due to the golden pompano rearing condition, age, sex, muscle composition, and nutrition of the feed, respectively. The fat level of hybrid grouper was much lower than silver pomfret (Xu et al., 2012).

Different groups usually categorize fish according to their crude fat contents, such as lean muscle (<2%), the low‐fat muscle (2%–4%), medium‐fat muscle (4%–8%), and high‐fat muscle (>8%) (Haard et al., 1994). In this present study, hybrid grouper and golden pompano could be categorized into lean‐fat to medium‐fat fish. The ash contents of golden pompano were significantly higher (*p* < .05) than the hybrid grouper.

### 3.3 Texture, color, and pH

Texture profiles including hardness, adhesion, elasticity, cohesion, chewiness, color, and pH parameters of golden pompano and hybrid grouper muscle are shown in Table 2. The textural values were found significantly different (*p* < .05) between golden pompano and hybrid grouper. The hardness values (206.89–377.7 N) of the golden pompano were significantly higher than the hybrid grouper (86.57–163.97 N). The hardness values of both fish were greater than European seabass (Schrama et al., 2022) and Atlantic mackerel (Cropotova et al., 2018). Similar data were also observed in adhesion and chewiness values among different muscle portions. On the other side, no significant differences (*p* > .05) were found in the elasticity and cohesion contents which were similar to the textural values of yellow grouper (Li et al., 2011). Generally, the texture profile depends on several factors such as collagen and fat contents which are directly correlated to muscle fiber density. Due to intensive microbiological and autolytic processes occurring during fish slaughtering, the muscle becomes softer and elastic to less extent, which ultimately causes the differences in texture profile (Zhang et al., 2019). The data obtained showed that the golden pompano contained better texture due to higher textural contents than the hybrid grouper.

**TABLE 2:** Texture, color, and pH characteristics of back and abdomen muscles of fresh golden pompano and hybrid grouper

Contents	Golden pompano		Hybrid grouper	
	Back	Abdomen	Back	Abdomen
Hardness (*N*)	206.89 ± 8.83^b^	377.77 ± 51.85^a^	163.97 ± 60.88^c^	86.57 ± 35.27^d^
Adhesion(*N* mm)	0.17 ± 0.03^a^	0.18 ± 0.02^a^	−4.29 ± 0.66^c^	−2.69 ± 1.02^b^
Elasticity (mm)	0.48 ± 0.07^c^	0.18 ± 0.24^d^	1.32 ± 0.40^b^	1.46 ± 0.39^a^
Cohesion	0.24 ± 0.01^c^	0.40 ± 0.05^b^	0.28 ± 0.33^c^	0.62 ± 0.39^a^
Chewiness (mJ)	17.43 ± 5.58^b^	23.97 ± 3.03^a^	3.02 ± 1.35^c^	1.01 ± 1.02^d^
L*	46.25 ± 1.18^b^	53.77 ± 1.04^a^	42.18 ± 1.5^c^	45.37 ± 2.45^b^
a*	1.26 ± 0.41^b^	2.43 ± 0.14^a^	0.78 ± 0.23^d^	0.87 ± 0.37^c^
b*	2.49 ± 0.28^d^	4.41 ± 0.10^c^	5.73 ± 0.51^b^	6.17 ± 0.09^a^
*W*	46.32 ± 1.21^b^	54.05 ± 1.05^a^	42.46 ± 1.53^c^	45.61 ± 2.25^b^
pH	6.62 ± 0.07^b^	6.76 ± 0.09^a^	6.24 ± 0.02^d^	6.31 ± 0.05^c^

*Note*: The data were determined as means ± SD from triplicate values. Different letters between the same row represent a significant difference (*p* < .05).

Color is considered a sensory attribute that determines the consumers' interest regarding the freshness quality of fish products. The colorimetric analysis of golden pompano and hybrid grouper muscles, that is, back and abdomen, was performed as shown in Table 2. In this current study, the lightness (L*) values (46.25–53.77) of the golden pompano muscles were significantly higher than the hybrid grouper muscles (*p* < .05). These results were in accordance with Chaijan et al. (2010), who reported that lightness (L*) was higher in the abdomen muscle. A higher lightness (L*) content contributes to the significant loss of water contents and protein denaturation during the post mortem process (Singh et al., 2022). The changes caused by the destruction of the heme ring in heme proteins were also responsible for the higher lightness (L*) values of muscle (Cavonius & Undeland, 2017). The redness (a*) values of the abdomen muscle were higher than that of the back muscle in both fish species and higher redness (a*) values were noticed in the golden pompano (*p* < .05). The greater redness (a*) color of golden pompano was in accordance with the higher myoglobin content in their muscles. These results agreed with the Frigate mackerel and catfish muscle values reported by Chaijan et al. (2013). The yellowness (b*) of the hybrid grouper was significantly greater than the golden pompano. While the whiteness (*W*) in the muscles of golden pompano was higher than the hybrid grouper. The greater b* value of the abdomen agreed with the higher carotenoid contents in this muscle. Generally, the differences in the color of fish muscle have been attributed to the oxidation of protein with hemo groups such as myoglobin and hemoglobin. Furthermore, muscle color might also be changed by several other factors such as lipid oxidation, storage time, temperature, and diet (Monteiro et al., 2021). The abdominal portion of each fish species has contributed the highest whiteness values in this study. The abdomen portion might be due to the higher value of total pigment content in this muscle.

The pH values of different muscle portions were in 6.24–6.76. The data obtained were similar to the giant grouper's previous results (Chyuan‐Yuan et al., 2018). The lowest pH of the back might be associated with the high amount of glycogen and accumulation of lactic acid in this muscle (Guo et al., 2021).

### 3.4 Water‐holding capacity

The cooking, drip, and water loss rates of different muscles of each fish species are shown in Figure 2b. The water‐holding capacity (WHC) is used as an indicator to determine the muscle freshness quality. The water is abundantly held inside the myofibrils, between the myofibril components, sarcolemma and myofibril, and muscle bundles and cells (Oswell et al., 2021). Water loss can cause damage to soluble protein and high retained WHC causes reduced protein breakdown (Nemova et al., 2021). From the results, a higher WHC was found in hybrid grouper than in golden pompano. Water losses in the golden pompano were significantly higher (*p* < .05) than hybrid grouper. No significant difference in cooking was found between golden pompano and hybrid grouper (*p* > .05). These results followed the water loss rate of grass carp reported by Zhang et al. (2021). Water loss in the back muscle was examined higher in both fish species. The changes in the loss rate of muscles might be attributed to the denaturation of protein structure which led to the excessive water loss in fish muscle. During the cooking process, the heating causes changes in myosin structure and shrinkage of myofibrils leading to more integration of protein structure which is mainly contributed to higher moisture loss (Lin et al., 2021).

### 3.5 Low‐field nuclear magnetic resonance

The low‐field nuclear magnetic resonance (LF‐NMR) is a safe testing technique often used to determine moisture particle movement and their accessibility in food samples (Duflot et al., 2019). Water distribution of golden pompano and hybrid grouper in different muscle portions was investigated by LF‐NMR (Figure 2c). The data showed that three peaks represent three different water‐holding groups such as T_21_, T_22_, and T_23_ in the muscles of each fish species. T_21_ with a relaxation time between 0.1 and 1 ms represents strongly bound water was described as water binds very strongly to the macromolecules, T_22_ with a relaxation time between 10 and 100 ms described as immobilized water, strongly bound within the dense network of myofibrillar proteins while T_23_ with a relaxation time between 100 and 1000 ms shows free water binds externally to the myofibrillar protein base, respectively (Du et al., 2020).

The data obtained showed that the T_21_ relaxation time among all samples in both fish fluctuated from 10.27 to 12.9 ms, respectively, with no significant differences (*p* > .05). These results were related to the previous study of golden pompano reported by Sun et al. (2021). This could be due to the bound water being tightly bound to muscle proteins, which shows that bound water was not affected by any mechanical stress or microstructural change in muscle tissue (Cai et al., 2019). Conversely, T_22_ relaxation time in the golden pompano muscles (296.64–358.71 ms) was slightly significantly longer than that in the muscles of hybrid grouper (286.63–358.70 ms), illustrated that the binding capacity of immobilized water in muscle tissues became weaker and water mobility increased (Duflot et al., 2019), which agrees with their WHC results, respectively. This result suggested that the intracellular water moved to extracellular space that causes high intracellular ion concentration which may lead to aggregation, denaturation of protein molecules, cell membrane damage, and reduced immobilized water‐holding capacity (Sun et al., 2019). On the other side, the trend in the change of relaxation time of free water T_23_ was not significantly different, which indicates the minimum immobilized water was converted into free water. This result was related to the previous study of golden pompano investigated by Yang et al. (2022). The water distribution or mobility was mainly caused by the change of immobilized water that occurred in the myofibrillar structure of the muscle tissues (Li et al., 2019). Therefore, golden pompano had higher water distribution or loss than that of hybrid grouper.

### 3.6 Electron microscopy analysis

**FIGURE 3:**
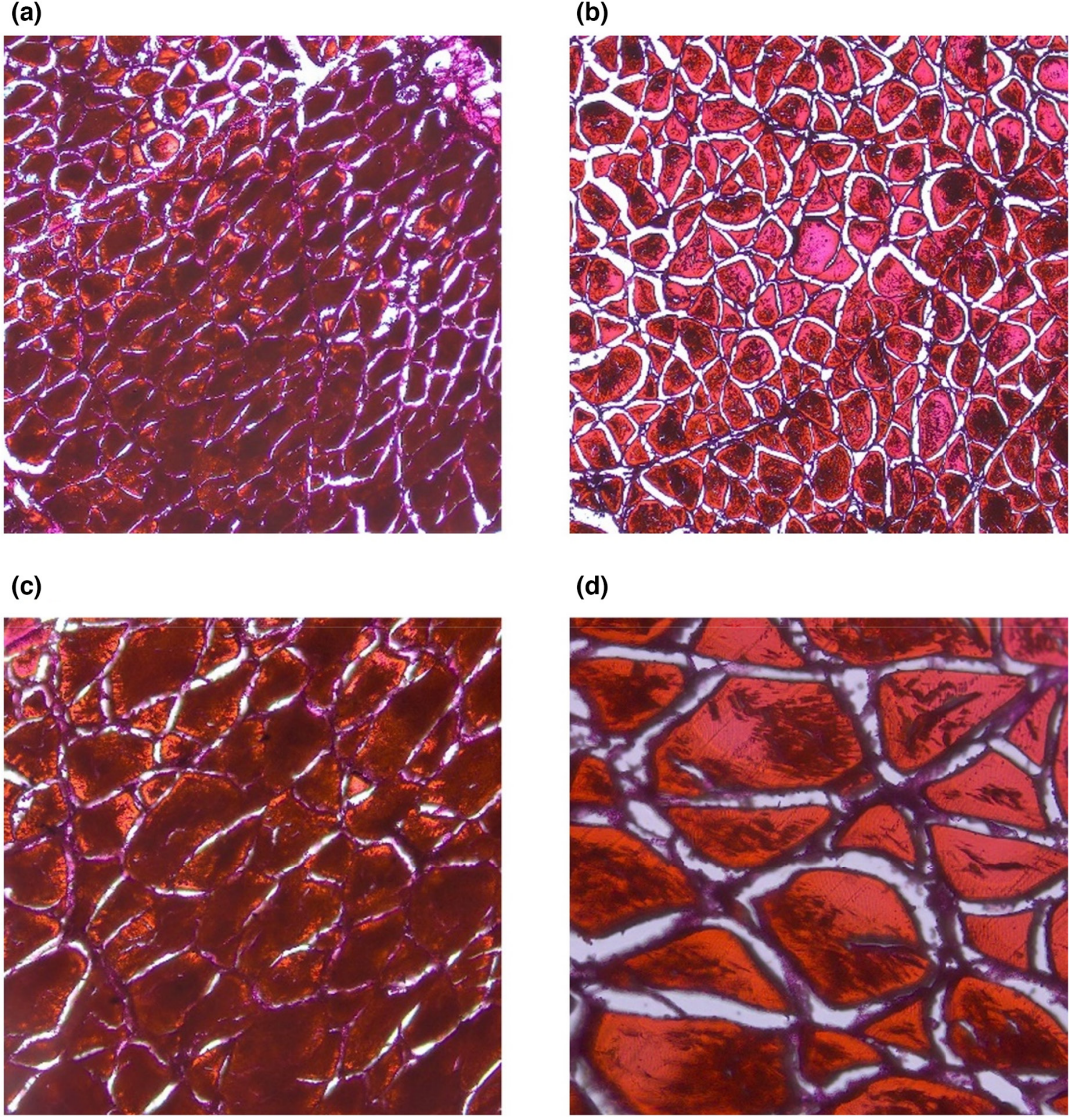
Cross‐section microscopic structure of (a) golden pompano (×200), (b) hybrid grouper magnification (×200), (c) golden pompano (×400), and (d) hybrid grouper magnification (×400).

The histological study of muscle fibers was performed and analyzed under the microstructure as shown in Figure 3. The muscle fiber cross‐sections of the two fish were observed under 10 × 20 and 20 × 20 magnifications, respectively. It was found that the muscle morphology and structure of both fish had a complete and dense muscle fiber microstructure. The cross‐section of the golden pompano was oblong, irregularly short, and circular. Even so, previous studies on various fish species have found that the diameter of muscle fibers was negatively correlated with the hardness of fish meat. The smaller the diameter of muscle fibers, the thinner the muscle fibers and greater the density of muscle fibers. Moreover, the firmness was higher and the fish was firmer and tastes better (Alomar et al., 2021). However, the cross‐section of the hybrid grouper was presented as a large irregular circle, oval, small triangular, relatively loose arrangement, and the intermuscular space was large. The intermuscular space was mainly the fat and connective tissue removed in the dehydration treatment because the adipocytes are rich in lipid droplets and the cytoplasmic substances were destroyed during the staining process and cannot be stained so the transparent gap appeared (Otto et al., 2006).

### 3.7 Protein composition

Proteins in the back and abdomen muscles of golden pompano and hybrid grouper were classified into four fractions according to their concentration as shown in Figure 2d. The protein contents were found significantly different (*p* < .05) in both the back and abdomen muscles. Overall, salt‐soluble protein constituted the major component (13.11–20.6 mg/g) in each muscle part and followed by water‐soluble protein (10.7–14.44 mg/g), salt‐insoluble protein (5.68–8.93 mg/g), and non‐nitrogenous protein (3.03–5.37 mg/g), respectively. These values were following the results of sardine and mackerel species where the salt‐soluble protein was the major component (2.39–28.46 mg/g) (Chaijan et al., 2004). The salt‐soluble protein contents of golden pompano were greater than the hybrid grouper. In both fish, the content of salt soluble in the abdomen was generally greater than in the back, respectively.

Additionally, the salt‐soluble protein was mostly involved in muscle contraction and contributed to the formation of fish gel. In this study, salt‐soluble protein values were greater than the large yellow croaker (Wei et al., 2016) and lower than that of common carp (Hao et al., 2021). The water‐soluble proteins were the second most dominant proteins, which were greater than salt‐insoluble and non‐nitrogenous proteins. A similar result was also found in pelagic fish species, that is, sardine and mackerel. Golden pompano had a larger amount of water‐soluble protein content than hybrid grouper, especially the abdomen muscle which had also been found in the farm‐raised catfish muscle (Chaijan et al., 2013). It has been suggested that the water‐soluble protein content in fish muscle can be increased due to excessive accumulation of myoglobin, enzymes, and other albumin proteins (Cai & Wang, 2021). On the other side, hybrid grouper contained a slightly greater amount of salt‐insoluble protein which could be possible due to the higher mechanical strength and accumulation of a larger amount of denatured protein in this fish (Bregeon et al., 2021). This result was also similar to the protein contents of sardine (Chaijan et al., 2004). The non‐nitrogenous protein content in golden pompano was slightly higher than the hybrid grouper. Overall, the golden pompano contained a higher amount of protein fractions and had shown high nutritional quality. This could be occurred due to increased contents of nucleotide, amino acids, urea, trimethylamine oxide, and dipeptides (Chaijan et al., 2013).

### 3.8 Minerals and heavy metal composition

As shown in Table 3, significant differences (*p* < .05) were found between two edible parts of the golden pompano and hybrid grouper muscles concerning their mineral composition. The differences in the mineral composition of fish muscles could be attributed to seasonal, biological, and other ambient factors (Fischer et al., 2021). Both fish back and abdomen muscles have been a great source of minerals and heavy metals. The essential minerals are involved in the proper functioning of acid–base equilibrium, skeletal structure, and maintenance of hormones, enzymes, and enzyme activators (Lall & Kaushik, 2021). They maintain the regularity of body fluid and cellular metabolism of the human body (Gupta & Gupta, 2014). Overall, the present study has found that potassium K was the major mineral (3700.56–4495.57 μg/g) followed by phosphorus P (2057.51–2704.07 μg/g), sodium Na (327.83–388 μg/g), and magnesium Mg (254.1–326.67 μg/g) in all muscle portions. Considering the K, Na, Mg, and Ca contents of hybrid grouper was found higher than the golden pompano. However, phosphorus content was significantly higher in the golden pompano than hybrid grouper (*p* < .05), respectively. These results were similar to that of aquacultured sea fishes (Erkan & Özden, 2007). Na and K help to improve energy metabolism, cell membrane permeability, maintenance of nerve, and normal cell function in the human body (Vilcanqui et al., 2021). Previously, Martins et al. (2017) studied mineral composition in pirarucu (*Arapaima gigas*) and reported similar results that the sodium and calcium contents were higher in the abdomen than back. This result was in accordance with the muscles of golden pompano.

**TABLE 3:** Mineral and heavy metal content in the back and abdomen muscles of fresh golden pompano and hybrid grouper

Elements	Golden pompano		Hybrid grouper	
	Back (μg/g)	Abdomen (μg/g)	Back (μg/g)	Abdomen (μg/g)
K	4396.33 ± 70.47^a^	3700.56 ± 78.83^a^	4495.57 ± 431.792^a^	3995.84 ± 239.37^a^
Na	327.83 ± 8.03^a^	342.27 ± 12.26^a^	388 ± 39.95^a^	364.67 ± 37.87^a^
Mg	306.81 ± 6.97^a^	254.1 ± 8.67^b^	326.67 ± 28.54^a^	286.67 ± 14.01^ab^
Ca	60.33 ± 6.22^b^	183.67 ± 91.29^a^	168 ± 59.23^ab^	131.86 ± 63.42^ab^
P	2704.07 ± 48.48^a^	2345.06 ± 74.7^b^	2271.57 ± 190.30^bc^	2057.51 ± 107.54^c^
Fe	6.76 ± 1.03^a^	7.63 ± 0.55^a^	3.62 ± 0.68^b^	3.71 ± 0.67^b^
Zn	5.86 ± 0.07^a^	6.06 ± 0.13^a^	4.49 ± 0.36^b^	3.9 ± 0.07^c^
Cu	0.39 ± 0.02^a^	0.44 ± 0.01^a^	0.23 ± 0.03^b^	0.26 ± 0.05^b^
Mn	0.11 ± 0.00^c^	0.12 ± 0.01^c^	0.18 ± 0.01^a^	0.16 ± 0.0^b^
Se	0.07 ± 0.01^b^	0.06 ± 0.01^b^	0.25 ± 0.11^a^	0.22 ± 0.04^a^
As	1.02 ± 0.00^a^	0.99 ± 0.02^a^	0.71 ± 0.02^b^	0.66 ± 0.12^b^
Pb (according to standard)	<0.03^a^	<0.03^a^	<0.03^a^	<0.03^a^
Cd (according to standard)	<0.03^a^	<0.03^a^	<0.03^a^	<0.03^a^
Hg (according to standard)	ND	ND	0.01 ± 0^a^	0.01 ± 0^a^

*Note*: The data were determined as means ± SD from triplicate values. Different letters within the row represent a significant difference (*p* < .05).

Abbreviation: ND, not detected.

The contents of heavy metals in the muscles of golden pompano and hybrid grouper were shown significant differences (*p* > .05) as presented in Table 3. The data obtained showed that iron Fe was the major component in all muscles and the content was found to be in the range 3.62–7.63 μg/g, followed by zinc (Zn) 3.9–6.06 μg/g and copper (Cu) 0.23–0.44 μg/g, respectively. Golden pompano showed higher contents of Fe, Zn, Cu, and arsenic (As) than hybrid grouper while the contents of manganese Mn and selenium Se were higher in the muscles of hybrid grouper. In this study, the contents of five constant elements of golden pompano are ranked in the following order: K > Na > Mg > Ca and P and the major trace elements were classified as Fe > Zn > Cu > Mn > Se, respectively. The content of other heavy metals including Pb, Cd, and Hg in different muscles meets national standards and there is no risk of excess heavy metals when consumed. The data showed that golden pompano and hybrid grouper are good sources of minerals. The amounts of heavy metals are considered negligible which shows that both fish species are rich sources of nutrients and have high eatable quality.

### 3.9 Fatty acids composition

The fatty acid composition of golden pompano and hybrid grouper muscles is shown in Table 4. Overall, 27 fatty acids were examined in both fish muscles, of which 11 were saturated fatty acids (SFA), 5 were monounsaturated fatty acids (MUFAs), and 11 were polyunsaturated fatty acids (PUFA). The fatty acid composition was varied significantly among different muscle portions. Among these, MUFAs were the most predominant fatty acids followed by SFA and PUFA, respectively. Similar fatty acid profiles in Asian catfish were also reported (Thammapat et al., 2010). MUFAs are an important factor used as a primary energy source in the development of fish growth and metabolism. SFAs are involved in the energy metabolism of fish species, therefore it shows that maximum usage of SFAs is not beneficial to human health, which can increase the risk of cardiovascular diseases (Liu et al., 2021).

**TABLE 4:** Fatty acid compositions of back and abdomen muscles of fresh golden pompano and hybrid grouper

Fatty acids	Golden pompano		Hybrid grouper		Fatty acids	Golden pompano		Hybrid grouper	
	Back (%)	Abdomen (%)	Back (%)	Abdomen (%)		Back (%)	Abdomen (%)	Back (%)	Abdomen (%)
C12:0	0.06 ± 0.00^a^	0.05 ± 0.00^b^	0.04 ± 0.00^b^	0.05 ± 0.00^b^	C18:4	0.32 ± 0.01^c^	0.29 ± 0.01^d^	0.49 ± 0.19^b^	0.93 ± 0.19^a^
C14:0	2.9 ± 0.01^b^	2.75 ± 0.01^c^	2.26 ± 0.43^d^	3.32 ± 0.38^a^	C20:2 n‐6	1.16 ± 0.04^b^	1.27 ± 0.04^a^	0.4 ± 0.18^d^	0.66 ± 0.08^c^
C15:0	0.29 ± 0.00^b^	0.2 ± 0.10^c^	0.21 ± 0.06^c^	0.34 ± 0.04^a^	C20:3 n‐3	0.55 ± 0.01^a^	0.54 ± 0.02^a^	0.09 ± 0.00^c^	0.12 ± 0.00^b^
C16:0	23.42 ± 0.32^b^	23.1 ± 0.25^c^	24.17 ± 6.09^a^	16.51 ± 1.89^d^	C20:3 n‐6	0.21 ± 0.00^a^	0.18 ± 0.00^b^	0.05 ± 0.01^d^	0.1 ± 0.01^c^
C17:0	0.23 ± 0.00^c^	0.21 ± 0.00^c^	0.24 ± 0.05^b^	0.33 ± 0.02^a^	C20:4	0.33 ± 0.01^b^	0.31 ± 0.03^c^	0.32 ± 0.14^b^	0.64 ± 0.13^a^
C18:0	4.89 ± 0.04^c^	4.56 ± 0^d^	11.41 ± 4.81^a^	6.01 ± 0.8^b^	C20:5 n‐3 (EPA)	0.86 ± 0.04^c^	0.77 ± 0.04^d^	2.74 ± 0.75^b^	4.57 ± 0.87^a^
C20:0	0.17 ± 0.01^b^	0.15 ± 0.01^c^	0.16 ± 0.04^c^	0.22 ± 0.03^a^	C22:5 n‐3	1.21 ± 0.02^b^	1.16 ± 0.05^c^	0.77 ± 0.36^d^	1.28 ± 0.2^a^
C22:0	0.12 ± 0.00^a^	0.11 ± 0.00^a^	0.12 ± 0.03^a^	0.13 ± 0.02^a^	C22:5 n‐6	0.22 ± 0.01^a^	0.21 ± 0.02^a^	0.07 ± 0.03^c^	0.1 ± 0.03^b^
C23:0	0.01 ± 0.00^a^	0.01 ± 0.00^a^	0.02 ± 0.00^a^	0.02 ± 0.00^a^	C22:6 n‐3 (DHA)	4.74^d^	5.26^c^	8.72 ± 2.04^b^	11.39^b^
C24:0	0.05 ± 0.00^c^	0.05 ± 0.00^c^	0.11 ± 0.01^a^	0.08 ± 0.00^b^	SFAs	32.20	31.24	39.61	28.33
C16:1	5.47 ± 0.04^a^	5.28 ± 0.02^b^	2.97 ± 0.79^d^	4.33 ± 0.42^c^	MUFAs	37.63	37.28	24.34	25.56
C18:1	27.61 ± 0.22^a^	27.34 ± 0.28^b^	18.39 ± 2.03^d^	18.67 ± 1.18^c^	PUFAs	29.40	30.94	34.21	43.09
C18:1 *trans*	2.69 ± 0.01^b^	2.72 ± 0.01^a^	1.77 ± 0.4^d^	2.18 ± 0.14^c^	UFAs	67.03	68.23	58.55	68.65
C25:0	1.34 ± 0.02^a^	1.31 ± 0.01^b^	0.87 ± 0.35^c^	1.32 ± 0.15^b^	n‐3 PUFAs	7.36	7.73	12.8	18.29
C22:1	0.28 ± 0.01^d^	0.37 ± 0.03^c^	0.83 ± 0.38^b^	0.28 ± 0.05^a^	n‐6 PUFAs	21.24	22.51	21.4	24.8
C14:1	0.21 ± 0.01^a^	0.24 ± 0.01^a^	0.38 ± 0.22^a^	0.1 ± 0.02^a^	EPA + DHA	5.61	6.03	11.46	15.96
C18:2 n‐6	19.65 ± 0.12^d^	20.85 ± 0.14^b^	20.26 ± 3.18^c^	22.6 ± 0.78^a^	DHA/EPA	5.51	6.83	3.18	2.49
C18:3	0.1 ± 0^a^	0.08 ± 0.01^a^	0.3 ± 0.01^a^	0.7 ± 0.32^b^	n‐6/n‐3	2.95	2.96	1.67	1.36

*Note*: The data were determined as means ± SD from triplicate values.

The contents of MUFA were found to be in the range 24.34%–37.63% and showed the highest in the golden pompano. Among them, oleic acid (C18:1) and palmitoleic acid (C16:1) were the dominant MUFA in all muscle portions. Similar findings were also reported for sea bass (Alasalvar et al., 2002). A recent study has shown that MUFAs have various beneficial effects on the human body as they can inhibit the risk of cardiovascular disease and several other inflammatory‐associated diseases while the influence of different MUFAs could be different (Tsehay et al., 2021). The amounts of C18:1 and C16:1 were analyzed which differed significantly (*p* < .05) in all muscles, and both were highest in the golden pompano than hybrid grouper. The differences in fatty acid composition can be caused by several factors such as reproduction stage, age, sex, cultivation condition, and feeding (Ali et al., 2021). In this study, the saturated fatty acids content of the hybrid grouper (67.94%) was significantly higher than the golden pompano (63.44%). Among the SFAs, mainly palmitic acid (C16:0) and stearic acid (C18:0) were found to be the most abundant fatty acids in all muscles, especially higher in the back muscle studied. A similar result has also been described for the wild and farmed sea bass (Smichi et al., 2017). The content of PUFA in all muscles was found to be in the range 29.40%–43.09% and had shown the highest content in the hybrid grouper. PUFAs help to regulate prostaglandin synthesis and induce wound healing (Kedar, 2020). Furthermore, PUFAs have been considered less effective in significantly controlling cholesterol and hypertriglyceridemia compared to SFAs, whereas different unsaturated fatty acids could be different (Prato et al., 2018). Among n‐6 PUFA, it is examined that linoleic acid C18:2 (19.65%–22.6%) was the major fatty acid among the muscles and these data also agree with the results reported by Chan et al. (2019). The content of C18:2 fatty acid in the hybrid grouper was generally higher than in the golden pompano. The docosahexaenoic acid (DHA) C22:6 (4.74%–11.89%) and eicosapentaenoic acid (EPA) C20:5 (0.77%–4.57%) were found as the most abundant n‐3 PUFAs differed vary significantly (*p* < .05) between all muscle portions. Since the nutritionist has been focusing on the advantages of C18:2, EPA and DHA sources in the prevention of cardiovascular diseases and atherosclerosis (Panagiotakos & Kouvari, 2021). From both fish species, the abdomen muscle had a larger amount of DHA than back muscle which has also been reported in the muscles of Asian catfish (Thammapat et al., 2010). The DHA content was generally higher in the muscles of hybrid grouper fish species. A similar trend has also been examined for the higher EPA contents in hybrid grouper than golden pompano, respectively. Hybrid grouper muscle had a higher content of EPA + DHA (11.46%–15.96%) than had golden pompano muscle. These data are in accordance with the values of longfin batfish (Liu et al., 2019). The DHA/EPA values in both fish muscles ranged from 2.49% to 6.83%, indicating that these fatty acids of both fish species can be used to defend the cardiovascular diseases and strengthen the nutraceutical sector for the protection of the cardiovascular system. The results obtained showed that both fish species were significantly rich sources of essential fatty acids. Comparatively, golden pompano can be considered a good source of fatty acids due to the high content of n‐6/n‐3 PUFA than the hybrid grouper.

### 3.10 Amino acids composition

The amino acid profile in different muscles of golden pompano and hybrid grouper on fresh bases is presented in Table 5. About 18 amino acids were detected in the muscles of both fish species, of which 11 were EAA, including isoleucine Ile, leucine Leu, lysine Lys, methionine Met, phenylalanine Phe, threonine Thr, tryptophan Try, valine Val, cysteine Cys, and tyrosine Tyr, and 7 were non‐essential amino acids (NEAA), including alanine Ala, arginine Arg, aspartic acid Asp, glutamic acid Glu, glycine Gly, proline Pro, and serine Ser, respectively.

**TABLE 5:** Amino acid composition of the back and abdominal muscles of fresh golden pompano and hybrid grouper

Amino acids	Golden pompano		Hybrid grouper	
	Back (mg/g)	Abdomen (mg/g)	Back (mg/g)	Abdomen (mg/g)
His	5.31 ± 0.47^a^	4.65 ± 0.39^b^	2.38 ± 0.29^d^	2.6 ± 0.14^c^
Ile	7.71 ± 0.74^a^	6.81 ± 0.31^d^	6.95 ± 0.4^c^	7.19 ± 0.76^b^
Leu	14.56 ± 0.35^c^	13.84 ± 0.68^d^	15.21 ± 0.15^a^	14.94 ± 1.27^b^
Lys	18.08 ± 0.53^a^	16.64 ± 0.45^b^	11.73 ± 1.22^d^	12.23 ± 0.34^c^
Met	5.45 ± 0.10^a^	5.47 ± 0.27^a^	3.85 ± 1.01^b^	3.84 ± 0.98^b^
Phe	8.25 ± 0.35^c^	7.64 ± 0.32^d^	15.65 ± 0.36^b^	16.06 ± 1.39^a^
Thr	8.65 ± 0.14^b^	8.56 ± 0.40^c^	9.16 ± 0.33^a^	9.17 ± 0.47^a^
Trp	0.73 ± 0.07^c^	0.69 ± 0.11^d^	1.71 ± 0.73^a^	1.62 ± 0.63^b^
Val	8.22 ± 0.63^a^	7.33 ± 0.29^b^	6.40 ± 0.57^d^	6.95 ± 0.48^c^
Tyr	5.70 ± 0.25^c^	5.39 ± 0.28^d^	5.81 ± 0.18^b^	5.84 ± 0.31^a^
Cys	1.42 ± 0.20^a^	1.36 ± 0.05^b^	1.18 ± 0.18^c^	1.37 ± 0.19^b^
Ala	14.22 ± 1.62^a^	12.58 ± 0.59^c^	12.61 ± 0.9^b^	12.39 ± 0.28^d^
Arg	11.78 ± 0.55^a^	10.87 ± 0.52^c^	10.81 ± 0.72^d^	11.05 ± 0.41^b^
Asp	18.35 ± 0.61^a^	17.82 ± 0.72^b^	17.71 ± 1.04^c^	17.26 ± 0.88^d^
Glu	27.27 ± 0.78^c^	26.49 ± 1.65^d^	28.14 ± 1.41^a^	27.77 ± 1.51^b^
Gly	11.46 ± 1.5^c^	10.51 ± 0.56^d^	12.08 ± 1.47^b^	12.6 ± 1.02^a^
Pro	5.83 ± 0.16^c^	5.96 ± 0.07^a^	5.93 ± 0.59^b^	5.68 ± 0.1^d^
Ser	7.72 ± 0.19^a^	7.47 ± 0.30^b^	7.45 ± 0.48^c^	7.46 ± 0.33^c^
TAA	180.64	169.99	174.76	176.02
EAA	82.65	76.96	78.85	80.44
NEAA	97.99	93.03	95.91	95.58
DAA	71.27	67.40	70.55	70.04
EAA/TAA	0.84	0.83	0.45	0.46
EAA/NEAA	0.46	0.45	0.82	0.84
DAA/TAA	0.39	0.40	0.40	0.39

*Note*: The data were determined as means ± SD from triplicate values. Different letters within the row indicate significant differences (*p* < .05).

Abbreviations: TAA, total amino acid; EAA, essential amino acid; NEAA, non‐essential amino acid; DAA, delicious amino acid.

Nonetheless, the amino acid profiles of the golden pompano were different from the hybrid grouper. The major amino acids in all muscles were glutamic acid followed by aspartic acid, lysine, leucine, glycine, arginine, and phenylalanine with the lowest level of tryptophan, respectively. Similar results were reported in longfin batfish (Liu et al., 2019). Glutamine was considered a dominant amino acid in the human body making up 60% contribution in the amino acids' constituents of the skeletal muscle. During critical illness, glutamine efflux from muscle assists as a donor source of important ammonia to the immune system (Deutz et al., 1992). The glutamic acid contents of the hybrid grouper were higher in the golden pompano while aspartic acid contents were higher in the golden pompano. The following amino acids such as glutamic acid, alanine, aspartic acid, and glycine contribute to 39.45% and 39.63% and 40.36% and 39.78% of the total protein in both fish muscles, respectively. Hybrid grouper had collectively the highest percentage of these amino acids compared with the golden pompano which was 40% higher than the recommended value, giving hybrid grouper a more delicious taste (Liu et al., 2019). The amounts of total amino acids (TAA) in the back and abdomen muscles varied from 180.64 to 169.99 mg/g in golden pompano and 174.76 to 176.02 mg/g in hybrid grouper.

Regarding the essential amino acids (EAA), the amounts in the back and abdomen muscles ranged from 76.96 to 82.65 mg/g in golden pompano and 78.85 to 80.44 mg/g in hybrid grouper. TAA contents in the hybrid grouper were significantly higher (*p* < .05) than those in the golden pompano muscles, while the contents of EAA in golden pompano were higher than hybrid grouper. These values were also higher than the yellowfin tuna and bigeye tuna (Peng et al., 2013). The EAA/TAA ratios in the muscles were found similar in both marine fishes which are well comparable to the reference value of 0.40 recommended by FAO/WHO (FAO/WHO, 1991). These findings agreed with earlier reports in the red tail (Jiang et al., 2017). The amounts of the non‐essential amino acid (NEAA) in all muscles varied from 93.03% to 97.99% in the golden pompano and 95.58% to 95.91% in the hybrid grouper. However, the delicious amino acid (DAA) contents in all muscles varied from 67.40% to 71.24% in golden pompano and 70.04% to 70.55% in hybrid grouper which are comparatively higher than the values in farmed pufferfish muscles reported by Tao et al. (2012). The amounts of NEAA in the hybrid grouper were slightly higher than in the golden pompano.

Similarly, the ratios of EAA/NEAA of hybrid grouper were found higher than those of the golden pompano muscles. These ratios were higher than the recommended value of 0.60 (FAO/WHO, 1991). Our results showed that the ratio of EAA/NEAA was comparable to the farmed pufferfish fishes such as *Fugu obscurus*, *F. flavidus*, and *F. rubripes* (Tao et al., 2012). Meanwhile, the contents of TAA and NEAA were found higher in the hybrid grouper while EAA and DAA contents were higher in the golden pompano, respectively.

The amino acid composition is an important factor employed to evaluate the nutritious value of protein, and the amino acid score AAS has widely been used for the assessment of protein quality. Many methods can be used to evaluate protein quality such as amino acid score (AAS), chemical score (CS), and essential amino acid index (EAAI). Thus, AAS is the percentage of amino acids in 1 g of protein sample divided by the recommended value of FAO/WHO reference amino acid scoring pattern. CS is the percentage of amino acids in 1 g of protein sample compared to the recommended 1 g of egg protein. EAAI method is used to compare the contents of EAA in a specific protein with the reference protein. If overall values of AAS or CS are >1, it shows that the target protein is rich in nutritional quality. The EAAI value of 0.95 or above indicates a high nutritional quality of the target protein. If the EAAI value lies between 0.85 and 0.95, it indicates that a target protein has good quality. If the EAAI value is between 0.75 and 0.86, which shows the nutritional quality of the target protein is only available and the EAAI score is below 0.75, it indicates the status of the target protein is not favorable (Shahidi & Ambigaipalan, 2018). The AAS, CS, and EAAI scores of golden pompano and hybrid grouper protein are presented in Table 6. According to the AAS, the amount of lysine (Lys) was the highest score while tryptophan (Trp) was described as the limited amino acid in all muscles. The AAS, CS, and EAAI of all EAA in the different parts of both fish species were above 1.00. These results indicated that the contents of essential amino acids in both fish species are well balanced and have high muscle protein quality.

**TABLE 6:** AAS, CS, and EAAI of back and abdomen muscle proteins of fresh golden pompano and hybrid grouper

Scores	Amino acids	Golden pompano		Hybrid grouper	
		Back	Abdomen	Back	Abdomen
AAS	Ile	0.91	0.79	2.78	2.88
	Leu	0.98	0.91	3.46	3.4
	Lys	1.58	1.42	3.45	3.6
	Met + Cys	0.92	0.9	2.29	2.37
	Phe + Tyr	1.09	0.99	5.65	5.77
	Thr	1.03	0.99	3.42	3.67
	Trp	0.34	0.32	2.85	2.71
	Val	0.79	0.69	2.06	2.24
CS	Ile	0.69	0.6	0.21	0.22
	Leu	0.81	0.75	0.28	0.28
	Lys	1.22	1.09	0.27	0.27
	Met + Cys	0.53	0.51	0.13	0.14
	Phe + Tyr	0.73	0.67	0.38	0.39
	Thr	0.88	0.85	0.29	0.31
	Trp	0.2	0.19	0.16	0.15
	Val	0.55	0.48	0.14	0.16
EAAI		2.08	1.6	2.33	2.59

*Note*: The data were determined as means ± SD from triplicate values.

## 4 CONCLUSIONS

This current study found significantly different proximate compositions in the back and abdomen muscles of both fish. Water losses and L* of golden pompano were significantly higher than the hybrid grouper which also agrees with the results of LF‐NMR. The textural profile of the golden pompano was better than the hybrid grouper due to the higher content of myofibrillar protein and pH values. Both n‐6 PUFAs and n‐3 PUFAs contents in golden pompano were greater than the hybrid grouper which indicated golden pompano was a good source of n‐3 PUFAs. The amino acid scores of all essential amino acids in both fish were greater than 1 which indicated that the essential amino acids in both fish species were well balanced and had high nutritional quality and are valuable food for human consumption. In conclusion, this present study provided valuable information on the physiological properties and nutritional composition of golden pompano and hybrid grouper, which would help researchers and industries to choose the right part or fish species for further food processing or research to obtain higher quality and availability. However, in the future, multiomics‐based technologies might be applied to evaluate the quality and monitor the process of marine foods, which will afford more specific and efficient information for researchers, industries, and consumers.

## CONFLICT OF INTEREST

The authors declare no conflict of interest.

## Data Availability

The data that support the findings of this study are available from the corresponding author upon reasonable request.
